# Loss of MAPK Pathway Activation in Post-Mitotic Retinal Cells as Mechanism in MEK Inhibition-Related Retinopathy in Cancer Patients

**DOI:** 10.1097/MD.0000000000003457

**Published:** 2016-05-06

**Authors:** Elon H. C. van Dijk, Danique E. M. Duits, Mieke Versluis, Gregrorius P. M. Luyten, Arthur A. B. Bergen, Ellen W. Kapiteijn, Mark J. de Lange, Camiel J. F. Boon, Pieter A. van der Velden

**Affiliations:** From the Department of Ophthalmology (EHCVD, DEMD, MV, GPML, MJDL, CJFB, PAVDV), Leiden University Medical Center, Leiden; Department of Ophthalmology (AABB); Department of Clinical Genetics (AABB), Academic Medical Center; Department of Clinical and Molecular Ophthalmogenetics (AABB), The Netherlands Institute for Neurosciences/Royal Netherlands Academy of Arts and Sciences, Amsterdam; and Department of Medical Oncology (EWK), Leiden University Medical Center, Leiden, the Netherlands.

## Abstract

Recently, treatment with MEK inhibitors has been shown to be an effective treatment option for metastatic melanoma. Treatment efficacy is dependent on inhibition of MAPK-related melanoma proliferation. However, targeting of MEK can be accompanied by a time-dependent and reversible serous retinopathy of unknown origin.

We analyzed the molecular mechanism by which the MEK inhibitor binimetinib may lead to retinopathy, using neuroretina and cell models of retinal pigment epithelium (RPE).

Binimetinib inhibited the MAPK pathway while discontinuation of treatment resulted in reactivation. However, cell proliferation was not inhibited correspondingly during binimetinib treatment of ARPE19 cells. Remarkably, post-mitotic neuroretinal tissue displayed a strong MAPK activation that was lost after binimetinib treatment.

We propose that binimetinib-associated retinopathy is correlated with inhibition of the MAPK pathway in multiple retinal components. Retinal cells are able to regain the activation after binimetinib treatment, mimicking the reversibility of the retinopathy. As most retinal cells are nonregenerating, other mechanisms than stimulation of proliferation must be involved.

## INTRODUCTION

**FIGURE 1:**
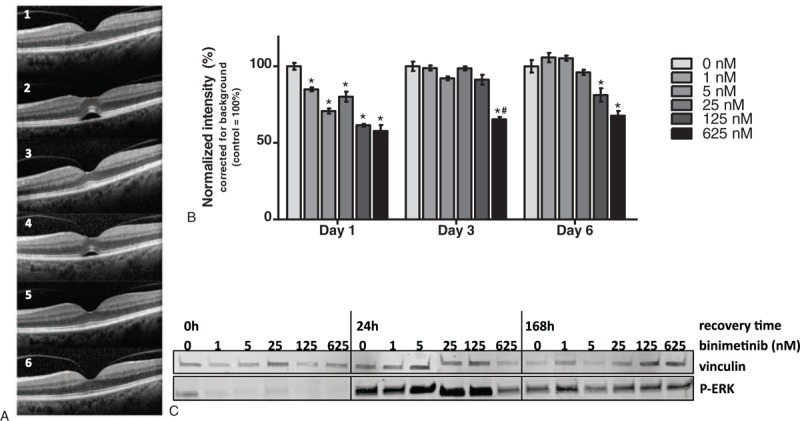
Illustration of the reversibility of binimetinib-associated serous retinopathy. (A) Ophthalmic imaging of the retina during binimetinib treatment of a 56-year-old female with metastatic cutaneous melanoma. At initial screening, the patient had a normal overall arrangement of the retinal layers (1). Eleven days after start of treatment an accumulation of subretinal fluid (SRF) had developed (2). Besides seeing dark flecks, the patient did not have ocular complaints. Thirty days after treatment started, SRF had disappeared despite continuation of medication (3). After discontinuation and restart of medication recurrence of SRF occurred (4), and (permanent) disappearance of SRF could be detected upon discontinuation of treatment due to a bad physical condition of the patient (5, 6). (B) Mild growth inhibition of ARPE19 cells upon increasing concentrations of binimetinib. Bars represent mean with SEM, control (0 nM) was set to 100%, and the intensities were corrected for background. ^∗^*P* < 0.05 compared to control (0 nM), and ^#^*P* < 0.05 compared to treatment with 125 nM. (C) Regain in ERK phosphorylation after 24 and 168 hours of recovery after treatment of ARPE19 cells with binimetinib. Both during treatment and recovery, vinculin expression was stable. ERK = extracellular signal-regulated kinase, SEM = standard error of the mean, SRF = subretinal fluid.

Inhibitors of MEK, such as binimetinib, have proven to be an effective treatment for patients with metastatic melanoma.^1^ However, serous retinopathy is a common complication of binimetinib.^1–4^ Binimetinib-associated serous retinopathy develops in up to 77% of metastatic cutaneous melanoma patients and in 60% of metastatic uveal melanoma patients.^2^ Although the central retina (macula) was affected in most patients, only 22% of all patients in our previous clinical study developed visual complaints. Dose reduction or discontinuation of the treatment with binimetinib led to the disappearance of complaints and subretinal fluid (SRF) in most patients. The symptoms recurred in most cases after restarting the treatment (Figure 1A). However, despite continuation of administration of binimetinib in the other patients, resolution of both complaints and lesions occurred.^2^ Electro-oculography, which is an indirect measurement of RPE cell function, was found to be abnormal in virtually all cases over a prolonged period. This indicates that binimetinib may induce persistent panretinal RPE cell dysfunction, despite resolution of SRF.^2,3^

Other MEK inhibitors such as trametinib, cobimetinib, and RO5126766 can also cause a similar retinopathy. This suggests that the development of retinopathy is not restricted to binimetinib, but related to the class of MEK inhibitors.^4–8^ This retinopathy is thereby most likely to be an on-target effect of MEK inhibition and may reflect the treatment efficacy. However, no relationship between occurrence of serous retinopathy and both overall survival and progression-free survival of cutaneous melanoma patients has been found.^2^ Despite the fact that activation of the MAPK pathway has only been observed in pathologic RPE, binimetinib-associated serous retinopathy occurs in eyes without a history of other ophthalmological diseases.^1^ A possible role for the MAPK pathway in the neuroretina has not been described yet. A better understanding of the underlying molecular mechanisms of the treatment enables the improvement of risk estimation of therapeutic effect in nonpathologic tissues. Therefore, we set out to analyze MAPK activation in RPE and neuroretina as a possible mechanism by which binimetinib causes serous retinopathy.

## MATERIALS AND METHODS

### Cell Culture

ARPE19 cells were cultured in order to serve as a positive control, exhibiting MAPK activity.^9^ Cells were cultured in DMEM/F-12 plus GlutaMax medium (Life Technologies, Carlsbad, CA) supplemented with fetal calf serum (Greiner Bio-One, Kremsmünster, Austria), penicillin, streptomycin, and glutamine (all Life Technologies) at 37°C with 5% CO_2_ at humidified conditions.

To obtain primary neuroretinal tissue, human tumor eyes were used to perform a separation of the neuroretinal layer and the choroid–RPE complex. A healthy part of an enucleated tumor eye was transferred to a CO_2_-independent medium (Life Technologies) to harvest the neuroretinal layer. After harvesting, the neuroretina was divided into 4 pieces and transferred to a 0.4 μm Corning Transwell polycarbonate membrane insert (Sigma Aldrich, St. Louis, MI), already prepared with a drop of CO_2_-independent medium. Under each insert 2 mL neurobasal-A medium (Life Technologies) with penicillin, streptomycin, glutamine, and B27 supplement (Life Technologies) was added. Before starting the experiment, the tissue cultures were maintained at 37°C in 5% CO_2_ at humidified conditions for at least 24 hours to allow the cells to recover from the dissection.^10^ The collection of material for research had been agreed upon by the Medical Ethics Committee of the Leiden University Medical Center (Leiden, the Netherlands) and the research protocol adhered to Dutch law and the current version of the tenets of the Declaration of Helsinki.

### Experiments

An in-cell-western assay was performed to analyze the effect of binimetinib (MW 441.23 g/mole) on cell proliferation of dividing ARPE19 cells. Besides the DMEM/F-12 plus GlutaMax medium with supplements as a control, the binimetinib dosages 1, 5, 25, 125, and 625 nM were tested. Following 24, 72, and 144 hours of treatment, the cells were fixed with 4% formaldehyde and DRAQ5 (Biostatus, Shepshed, UK) was used to stain the fixed cells. Intensity of DRAQ5 fluorescence was quantified with the Licor Odyssey infrared imaging system (LI-COR, Lincoln, NE). In an other experiment, ARPE19 cells were treated with binimetinib for 24 hours with the concentrations above. After treatment, binimetinib was replaced by the normal culture medium in order to allow the cells to recover for 24 or 168 hours.

After culturing the primary neuroretinal tissue, the medium was fortified with binimetinib, which was added to the wells under the inserts. The neuroretinal tissue was treated with 5, 25, and 125 nM binimetinib for 24 hours.

Following binimetinib treatment, protein of ARPE19 cells and of the pieces of primary neuroretina was isolated by using mammalian protein extraction reagent buffer supplemented with phosphatase and protease inhibitors (Life Technologies). Ten micrograms of protein was loaded on a mini-protean TGX gel (4–15%, Bio-Rad, Hercules, CA) with 10 wells, followed by transfer to a PVDF (low-fluorescence) membrane using the trans-blot turbo system (Bio-Rad). The primary antibodies used included mouse-anti-phospho-p44/42 ERK antibody (Sigma Aldrich), rabbit-anti-p44/42 ERK antibody (Cell Signalling Technologies, Danvers, MA), mouse-anti-vinculin antibody (Sigma Aldrich), and rabbit-anti-calbindin-D28K antibody (Cell Signalling Technologies). The secondary antibodies used were goat–anti-mouse 800 nm antibody and goat–anti-rabbit 680 nm antibody (LI-COR). The fluorescence intensity was measured using the Licor Odyssey infrared imaging system.

### Statistical Analysis

Statistical analysis of the results was performed in SPSS Statistics (IBM, version 23.0) using a nonparametric Mann–Whitney test to determine significance between different binimetinib concentrations. *P*-values <0.05 were considered to be statistically significant.

## RESULTS

Although no significant growth inhibition was observed (Figure 1B), ERK activation in ARPE19 cells decreased with increasing binimetinib concentrations (Figure 1C). Expression of total ERK was not affected by binimetinib treatment (data not shown), but a complete loss of ERK activation was observed.

The analysis of the reversible nature of the binimetinib-associated serous retinopathy in the ARPE19 cell line showed a decrease in ERK activity after 24 hours of binimetinib treatment with increasing concentrations (Figure 1C). Both 24 and 168 hours after discontinuation of binimetinib treatment, an increase in ERK activity was found, compared to immediately after the cessation of binimetinib treatment. The increase in ERK activity was similar for all concentrations of binimetinib (Figure 1C). This experiment showed the ability of ARPE19 to recover from binimetinib treatment within 24 hours.

**FIGURE 2:**
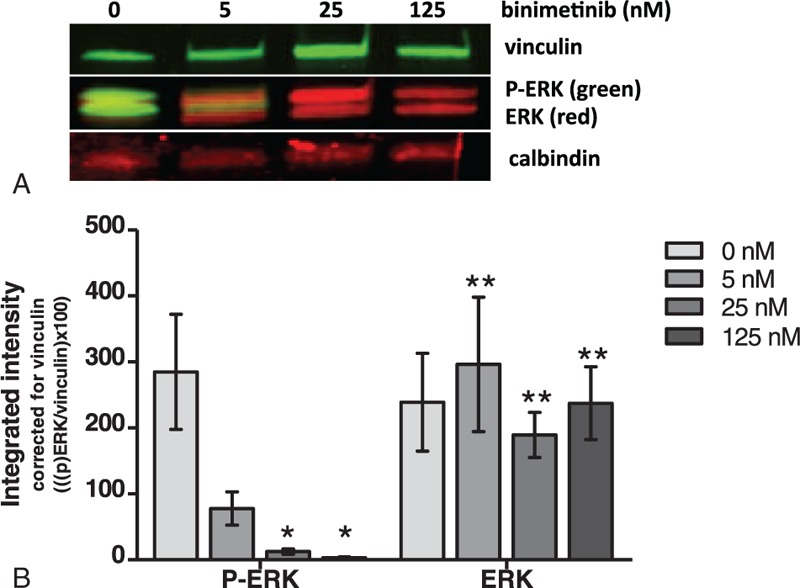
ERK activation in nonregenerative primary neuroretinal tissue. (A) Binimetinib reduced ERK activity (P-ERK) in primary neuroretinal tissue. MEK inhibition had no effect on the expression of calbindin, vinculin, and total ERK. (B) In all 7 neuroretinas, a reduction in ERK activity was observed after treatment with binimetinib. The differences in activity after treatment with binimetinib concentrations of 25 and 125 nM, when comparing to 0 nM, were significant (*P* = 0.004 and *P* < 0.001, respectively). Between the binimetinib concentrations, no significant difference was seen in total ERK expression. The integrated intensities of both ERK and total ERK were corrected for vinculin ([{p}ERK/vinculin] × 100). Bars represent mean with SEM. ^∗^*P* < 0.05 compared to control (0 nM), and ^∗∗^*P* > 0.05 compared to control (0 nM). ERK = extracellular signal-regulated kinase, MEK = mitogen-activated protein kinase kinase, SEM = standard error of the mean.

Primary neuroretinal tissue, unexpectedly, displayed activated ERK (Figure 2A). A reduction in ERK activity was observed in all 7 tested neuroretinas following binimetinib treatment with increasing concentrations (Figure 2A and B). Binimetinib treatment at 25 and 125 nM significantly decreased ERK activity (*P* = 0.004 and *P* < 0.001, respectively). Calbindin antibody was used to validate the presence of neuroretinal tissue in all 7 tested samples by staining the ganglion cell layer, the inner nuclear layer, and the outer nuclear layer. Calbindin and total ERK expression were not affected by binimetinib treatment (Figure 2A and B).

## DISCUSSION

Based on the results of this study, we propose that the binimetinib-associated serous retinopathy represents a class effect of MEK inhibitors and that it therefore is most likely to be an on-target side effect. This leads to the challenging hypothesis that MAPK activation is playing a role in the retina, an essentially nonregenerating tissue.

We analyzed MAPK activation in a RPE cell line model and in primary neuroretina to investigate involvement of MAPK inhibition in serous retinopathy. We revealed ERK activation in both ARPE19 and primary neuroretina. ERK activation in ARPE19 cells has been observed before,^11^ and gene expression analysis of primary RPE cells has suggested a broad functional role for ERK.^12^ ERK activation in neuroretina has not previously been described. Binimetinib treatment inhibits ERK in both the neuroretina and the RPE cell model. This potentially involves MAPK inhibition in the development of MEK-associated serous retinopathy. During recovery from the binimetinib treatment, ERK activation increased in the RPE model, at all concentrations. The in vitro ability to recover from treatment mirrors the disappearance of the serous retinopathy after treatment discontinuation. Upon resuming binimetinib treatment, serous retinopathy and visual symptoms often recurred. However, in some patients resolution of SRF occurred spontaneously despite continued treatment (Figure 1A).^2^ This time-dependent and self-limiting nature of the serous retinopathy suggests redundancy of ERK during prolonged MEK inhibitor treatment.

Binimetinib-treated patients display irreversible electro-oculography abnormalities, reflecting an impaired standing potential of the RPE and RPE dysfunction.^2,3^ Normally, the RPE prevents SRF accumulation by maintaining the outer blood-retinal barrier and by regulating ion channels.^13^ Tight junctions between the RPE cell monolayer that enable the RPE to form the outer blood-retinal barrier are regulated by the MAPK pathway.^14^ A factor that is involved in this process is the fluid transport channel aquaporin 1 that was specifically shown to be regulated by the MAPK pathway.^15^ The prolonged RPE dysfunction together with serous SRF accumulation that is observed in binimetinib treated patients may be explained by these mechanisms.

The clinical phenotype may also originate from abnormalities induced in the neuroretina, as we observed a strong activation of ERK in primary neuroretinal tissue. ERK is a neuroprotective regulator in the neuroretina and could be involved in the maintenance of a normal neuroretina-RPE interaction.^16,17^ ERK activity may also play a role in maintenance of the inner blood-retinal barrier that lines the retinal vasculature, by regulating tight junctions similar to the RPE outer blood-retinal barrier. Only few cases that developed a retinal vein occlusion during MEK inhibitor treatment have been described.^2,3,5,7,18^

Treatment consisting of both BRAF and MEK inhibitors improves efficacy in treating metastatic melanoma as the addition of a MEK inhibitor neutralizes the paradoxical MAPK pathway activation that occurs due to BRAF inhibition.^6^ For that reason, combination treatment reduces the risk of tumorigenesis in other tissues, such as the development of squamous cell carcinoma.^19^ However, the occurrence of a treatment-associated serous retinopathy increased with this combination, supporting the notion that this adverse event is most likely to be an on-target treatment effect of MAPK targeted drugs.^20^

In conclusion, MEK inhibitors such as binimetinib can cause a rapid-onset serous retinopathy in patients with metastatic melanoma, which is at least partially reversible. In monocular uveal melanoma patients, the functional visual impact may be clinically the most relevant. It is essential to unravel the (molecular) origin of this adverse event to be able to prevent it, for example, by means of a dose reduction in symptomatic patients.

Our findings show that the retinopathy may be caused by reversible suppression of ERK activity in neuroretina and RPE, which could lead to disturbances in the neuroretina–RPE interaction and to the occurrence of SRF. The strong activity of ERK in primary neuroretinal tissue indicates that ERK has other functions besides regulation of cell proliferation, as neuroretina represents post-mitotic neural tissue. Inhibition of retinal ERK activity appears to be a key component in the pathogenesis of MEK-associated serous retinopathy.
